# TMacaque-FaceNet: Automatic Facial Recognition Based on Vision Transformer for Wild Tibetan Macaques

**DOI:** 10.3390/ani16071107

**Published:** 2026-04-03

**Authors:** Qiyang Gao, Lele Zhang, He Luo, Zhao Lv, Dongpo Xia

**Affiliations:** 1 School of Life Sciences and Medical Engineering, Anhui University, Hefei 230601, China; 2 International Collaborative Research Center for Huangshan Biodiversity and Tibetan Macaque Behavioral Ecology, Hefei 230601, China; 3 School of Computer Science and Technology, Anhui University, Hefei 230601, China

**Keywords:** *Tibetan macaque*, individual recognition, vision transformer, YOLO

## Abstract

Within the framework of behavioral ecology and conservation, individual recognition plays a critical role in the research on wild social animals at the individual level. Traditional identification methods often rely on long-term field experience or invasive physical tagging. Recent advances in deep learning enable non-invasive individual recognition under natural conditions; however, the effectiveness of facial detection and identification depends on species-specific facial characteristics, environmental conditions, and dataset scale. In this study, we used 3385 images from 18 identified wild Tibetan macaques (*Macaca thibetana*) to develop an individual recognition system, TMacaque-FaceNet, integrating You Only Look Once (YOLO) for face detection and a Vision Transformer (ViT) for individual classification. The results showed that the Tibetan macaque face detector achieved a mAP@0.5 of 0.971, with a precision of 0.974 and a recall of 0.931. The individual recognizer for the wild Tibetan macaque social group achieved a top-1 accuracy of 96.33% on the test set. On an event-wise (temporal holdout) validation set comprising 90 images (5 images per individual), the recognizer achieved a top-1 accuracy of 95.56%. Gradient-weighted attention rollout analyses further revealed that the model focused on biologically meaningful facial regions, supporting the interpretability of the recognition process. Our results provide a new automated facial recognition method to non-invasively monitor Tibetan macaque individuals in natural environments. It provides a practical tool to facilitate automated behavioral observation, social network analysis, and long-term population monitoring of wild non-human primates.

## Simple Summary

Accurate individual identification is important for studying wild primates, but traditional methods are often time-consuming and invasive. In this study, we developed TMacaque-FaceNet, an automatic facial recognition framework for wild Tibetan macaques. Our results showed that TMacaque-FaceNet could effectively learn individual facial features and mainly rely on facial information for recognition. The framework also showed good adaptability to complex natural environments under small-sample conditions. This study provides a practical and non-invasive tool for the long-term monitoring of wild Tibetan macaques and may support future research on other similar primate species.

## 1. Introduction

Within the framework of behavioral ecology and conservation, particularly when focusing on wild social animals at the individual level, individual recognition is crucial [1]. It is fundamental for understanding complex phenomena such as social group dynamics, the transmission of characteristics across generations (i.e., trait inheritance across lineages), phenotypic changes that occur throughout an individual’s lifespan, and the dynamics of behavioral transmission and social relationships [2]. A growing body of research indicates that many fundamental questions can only be addressed through long-term studies of individually identifiable animals. Accurate individual identification provides a robust foundation for investigating temporal changes in population structure and for conducting quantitative genetic analyses of traits in wild populations [1]. It also enables the systematic documentation of individual variation in behavioral traits arising from genetic differences in social animals [2,3]. In a conservation context, individual identification is fundamental to the monitoring of the behavior and health status of specific individuals, thereby facilitating the development of more precise and effective conservation strategies [2,4,5].

Various methods have been employed for the identification and tracking of individuals, primarily involving artificial marking techniques such as ear tags, collars, dyes, and tattoos, which allow real-time identification of known individuals [6,7,8]. For species with distinctive and easily recognizable features, individuals can also be identified based on natural characteristics through the long-term experience of observers [5,9]. However, these methods still encounter several challenges in their practical application under field conditions in the wild. The deployment and maintenance of artificial markings typically require considerable resource investment, and some invasive marking techniques may induce physiological stress or behavioral disturbances, potentially affecting animal welfare and natural behavior [10,11,12]. Natural feature-based identification methods require extensive professional training and may be influenced by observer subjectivity, which can introduce inaccuracies. In addition, key identifying features may fade or change over time, complicating long-term individual recognition [13]. Consequently, the development of cost-effective and minimally invasive approaches for individual identification has emerged as a major challenge in contemporary ecological research. A range of non-contact identification techniques have also been increasingly adopted [14]. For example, in the conservation study of the Royal Bengal tigers (*Panthera tigris tigris*), researchers had collected DNA from feces, hair, and other remains of individuals, using molecular markers for genetic identification to determine their identities in wild environments [15]. This non-invasive molecular approach has substantially reduced disturbance to animals while ensuring high identification accuracy. However, its reliance on specialized equipment and laboratory processing has prevented real-time identification in the field, limiting the integration of behavioral observations with individual identity and thereby constraining its applicability.

With the development of field image acquisition technologies such as camera traps, large volumes of wildlife image data can now be obtained efficiently [16]. By combining these data with deep learning techniques, researchers can effectively process large-scale image datasets, construct datasets for individual identification, and enable automated and accurate individual identification based on phenotypic differences [17,18]. Compared with manual processing, deep learning methods are more efficient in handling large amounts of data and better able to capture complex underlying feature relationships. This advantage is particularly important for targets that are difficult to distinguish reliably with the naked eye, as deep learning can identify subtle yet informative phenotypic patterns. For example, Patton et al. [19] developed a photo-identification model for multiple cetacean species using an ArcFace-based deep learning architecture. The mean average precision (mAP) reached 0.869 on 21,192 test images. Among the various phenotypic traits used for automated recognition, the face contains rich identity-related information and has therefore become an important target for individual identification. This is especially relevant in primates, whose facial features often show substantial inter-individual variation [20]. Using the lightweight object detection model YOLOv8n, Paulet et al. [6] developed an individual recognition system for Japanese macaques (*Macaca fuscata*). The classification accuracy was 83%. AI-assisted identification methods have also been successfully applied to many primate species, including chimpanzees (*Pan troglodytes*) and Qinling golden snub-nosed monkeys (*Rhinopithecus roxellana*) [20,21]. Together, these studies demonstrate the strong potential of deep learning for primate individual recognition based on facial features.

AI-based individual identification methods continue to advance and evolve, yet they still face notable limitations in practical applications. First, accurate identification generally relies on large volumes of high-quality data. However, changes in the habitats of wild populations, such as altered migration patterns or environmental disturbances, can substantially disrupt fixed-location data acquisition. These interferences make it difficult to collect complete, high-quality datasets using methods such as camera traps [22]. Second, due to differences in species and task difficulty, as well as variations in dataset size, directly transferring an identification system from one population to another often degrades performance and may also lead to insufficient or inefficient use of computational resources. Therefore, it is essential to customize identification systems according to population size, species-specific characteristics, and environmental context [8]. Current research has largely focused on populations with well-established data collection systems and large-scale datasets, for which identification frameworks are becoming increasingly mature. However, the design of identification systems for populations with limited and difficult-to-acquire data remains underexplored. In this study, we aim to address this gap by improving data processing strategies to ensure more effective utilization of limited data, thereby establishing an identification system tailored to small-scale populations.

The Tibetan macaque (*Macaca thibetana*) is an endemic primate species in China, occupying a unique evolutionary position. It exhibits stable and distinguishable facial morphology with sparse facial fur and exposed skin regions, providing high-contrast landmarks for automated recognition [23]. Therefore, we have decided to use the Tibetan macaque YA1 group, residing in the Wild Monkey Valley of Mount Huangshan, as the subject of our research to establish an automated system for identifying wild Tibetan macaques (TMacaque-FaceNet), capable of detecting facial features of the macaques and automatically recognizing known individuals in natural field conditions, thereby providing a transferable framework for AI-assisted identification of other rare primate species.

## 2. Methods

### 2.1. Study Site and Subjects

**Figure 1:**
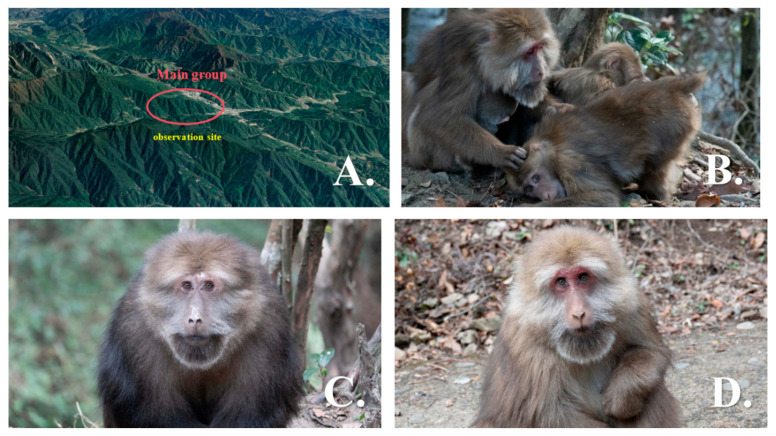
Contextual pictures of Mount Huangshan National Nature Reserve and its ‘main group’ population. (**A**) Satellite shot of Mount Huangshan National Nature Reserve (source: Google Earth). (**B**) ‘Main group’ macaques grooming each other. (**C**) Male individual image (**D**) Female individual image.

The study was conducted in the Mount Huangshan National Nature Reserve, Anhui Province, China, with a particular focus on the Yulinkeng A1 group (YA1). The Wild Monkey Valley (30°04′25″ N, 118°08′59.3″ E) is located at the foot of the Huangshan Scenic Area, at an altitude of 600–1200 m [24,25]. Since 1983, the YA1 group has been the subject of continuous, multi-generational behavioral and ecological research. Over more than four decades of long-term monitoring, each individual Tibetan macaque in the group has been systematically assigned a unique identification based on long-term observational records (Figure 1). The macaques in the study area live freely and independently, and their frequency of occurrence in the Wild Monkey Valley has increased in association with long-term supplementary feeding with corn.

### 2.2. Facial Data Collection

During the data collection period, the YA1 group was observed under the long-term provisioning regime consistently adopted by the research team for this study population. Dried maize was provided as supplementary food at 07:00, 14:00, and 18:00 each day. To minimize disturbance and avoid excessive aggregation and feeding competition, the maize was scattered across multiple locations on the ground within the Monkey Valley rather than concentrated at a single point. The amount of supplementary food varied according to the number of Tibetan macaques present in the valley on a given day but did not exceed one-third of the group’s daily food intake. This long-term standardized supplementary feeding protocol was intended to minimize disturbance to the Tibetan macaques.

Sampling was conducted according to the actual location of the study group. When the macaques entered the observation area, data collection was carried out there; when they were not present, we searched for them in the deeper forest and continued sampling in their natural activity area. Throughout filming, we maintained a shooting distance of more than 5 m whenever possible in order to minimize disturbance, and no deliberate interference with the animals’ ongoing behavior occurred during data collection. During data collection, we intentionally included samples with variation in facial angle, lighting condition, and shooting distance in order to better reflect the distribution of images encountered under real field conditions.

Video recording was conducted using a Sony RX10 (Sony Corporation, Tokyo, Japan). We employed a focal sampling method, in which each Tibetan macaque was tracked and filmed according to a pre-set order, combined with random sampling that prioritized individuals with fewer available samples. This sampling design increased the likelihood of capturing prolonged frontal views and diverse facial angles from the same individuals. Image data for this study were collected from July 2024 to March 2025, with sampling conducted daily from 07:00 to 18:00. A total of 2873 images and 5437 min of video data were collected through targeted tracking and random recording. The videos were reviewed using VLC Media Player 3.0.21 (VideoLAN, Paris, France), and high-quality facial images from multiple viewpoints were extracted for subsequent analysis.

### 2.3. Facial Image Compilation and Preprocessing

During the study period, the YA1 group included 19 adult Tibetan macaques, together with several subadult and juvenile individuals (Supplementary Table S1). Image data were collected for all 19 adults. However, one individual (TQG) had a very low appearance frequency during sampling. After video screening and image extraction, only 47 facial images were obtained for this individual, and these images showed limited temporal and event diversity. This sample was therefore considered insufficient to support reliable training and evaluation. Accordingly, TQG was excluded from the final dataset, which ultimately included 18 adult individuals.

After filtering the obtained images, we selected 3385 facial images from 18 individual macaques and classified them. The sample size per macaque ranged from 79 to 407 (Table 1). This variation was mainly related to differences in how often individuals entered the observation area and in the likelihood of obtaining clear facial images. Some macaques appeared less frequently, while others, due to individual behavioral tendencies, were more likely to sit in close clusters with group members, which often caused facial occlusion. To conduct both facial detection and individual identification experiments, we processed the dataset in two distinct ways. When splitting the dataset into training and test sets, we first ensured that the test images were extracted from different videos and remained independent from the training set in both temporal and event context, thereby reducing the risk of data leakage. For the training set, we made every effort to avoid selecting adjacent video frames. As far as possible, each image was selected either from an independent video or from a temporally distant segment within the same video.

In the facial detection experiment, all images were combined and randomly shuffled, then split into a training set and a test set at a ratio of 10:1. The facial regions in the images were manually annotated using the “Make Sense” application, and a category label file named “Monkeyface.txt” was created, with the images and labels placed in “image” and “label” folders, respectively, to serve as the dataset for training the facial detection model. For the individual identification experiment, the facial-detected images were again divided into training and test sets at a 10:1 ratio, with images from different individuals stored in separate folders (e.g., “001.bm”). To reduce potential individual bias introduced during manual classification, three researchers with extensive field experience in identifying individual Tibetan macaques from the YA1 group were invited to verify and score the data (with a maximum score of 100). Images with discrepancies in human identification were discussed collectively, and ambiguous images were removed. The remaining images were then randomly shuffled and re-scored, ensuring that the average score exceeded 95, to improve label reliability. The scoring results are summarized in Table 2.

### 2.4. Computational Setup

All experiments were conducted on a Linux (Ubuntu 22.04 LTS) workstation equipped with an NVIDIA RTX 4090 GPU with 24 GB of GDDR6X memory (using CUDA 12.8 for parallel acceleration) and a 64-core Intel Xeon CPU. The framework was implemented using PyTorch 2.8.0 (Python 3.12), with GPU memory optimization techniques such as mixed-precision training employed to support large-scale model training and inference.

### 2.5. Face Detection

Monkey face detection was trained using the Ultralytics implementation of YOLOv8s. The detector was initialized from COCO-pretrained weights (yolov8s.pt), with the backbone frozen, and fine-tuned on our dataset as a single-class detection task. Training was conducted for 100 epochs with a batch size of 16 and an input resolution of 1280 × 1280. We used the AdamW optimizer (initial learning rate 5 × 10^−4^, momentum 0.9, weight decay 1 × 10^−3^) with a cosine learning-rate schedule (lrf = 0.01). A 10-epoch warm-up was applied, and early stopping was enabled (patience = 25). Automatic mixed-precision training was used to accelerate optimization. To improve generalization, we applied standard data augmentations (geometric and photometric transforms) together with mosaic and Mixup; mosaic augmentation was disabled during the final 15 epochs to stabilize convergence.

### 2.6. Individual Identification

#### 2.6.1. Vision Transformer (ViT)

We used a Vision Transformer (ViT) as the primary architecture in this study. ViT models global image context via self-attention, which enables it to capture long-range dependencies among local regions. In addition, ViT offers flexibility in representing fine-grained details and overall structure, allowing it to emphasize key regional differences while preserving global contextual information—properties that are beneficial for fine-grained classification [26,27]. These advantages effectively address the challenges posed by the high similarity between the facial skin and fur coloration of Tibetan macaques, the low contrast in facial regions, and the blurred contours with a lack of distinct local features (e.g., color patches).

#### 2.6.2. Training Data Augmentation Module

The terrain in the Wild Monkey Valley of Huangshan is rugged, the vegetation is dense, and lighting conditions are unstable, making it extremely difficult to obtain high-quality facial image data. We implemented a series of targeted data augmentation techniques. These techniques are primarily categorized into spatial transformations, color perturbations, and mixed augmentations, aiming to enhance the model’s generalization ability and reduce the risk of overfitting.

In terms of spatial transformation, all training images were first resized to 1.1 times the input size (246 × 246 pixels) and were then randomly cropped to 224 × 224 pixels. This process introduced randomness in translation and scale while preserving the important image content. Horizontal flipping was applied with a probability of 0.5 to further augment the data. In addition, the ShiftScaleRotate transformation was used to simulate real-world variations in perspective and distance. With a probability of 0.5, a combination of translation (magnitude ≤ 8%), scaling (±10%), and rotation (±10 degrees) was applied. This not only generated new data samples but also simulated random behaviors of Tibetan macaques in the wild, such as head shifts during grooming or angular deviations and displacements when they searched for particles on themselves or others. By incorporating these geometric transformations, the study effectively expanded the limited training data while capturing the macaques’ natural movement patterns, thereby improving the model’ s practical applicability and generalization performance under field conditions.

With respect to color transformation, the lighting conditions in the Wild Monkey Valley of Huangshan were highly unstable, and the complex terrain resulted in both direct sunlight and large-scale occlusions. To address this issue, a series of color enhancement operations was performed. The first method was the ColorJitter transformation, which adjusted brightness (±15%), contrast (±15%), saturation (±15%), and hue (±5%). The second method was the HueSaturationValue transformation, which provided finer adjustments in color space, with a hue offset of 10 and saturation and brightness offsets of 15 and 10, respectively. These two strategies were randomly selected with a probability of 0.5. At the same time, the RandomBrightnessContrast transformation was applied, with a 0.4 probability of independently adjusting image brightness and contrast within the default range of ±15%. These color transformations effectively simulated the complex environmental conditions of the wild monkey valley and ensured that the model maintained high accuracy under diverse field environments. In addition, imaging degradation techniques were applied with a probability of 0.2, including Gaussian noise (variance 5–25) and Gaussian blur (kernel size 3–5), to simulate the decline in image quality during image acquisition and to enhance the model’s robustness when processing low-quality images.

For occlusion handling, block occlusion (Coarse Dropout) was employed with a probability of 0.3 to randomly mask 1 to 8 small regions. The size of each masked region was constrained to between 1/400 and 1/100 of the image area. This strategy forced the model to learn more robust local features and reduced its reliance on a single prominent feature. At the same time, it simulated partial occlusions caused by elements such as leaves and branches in natural environments, thereby enhancing the model’s practicality in field applications.

In terms of mixed data augmentation, an adaptive MixUp strategy was implemented based on the training process.

The MixUp operation can be formally expressed as:

(1) $$\tilde{x}=\lambda x_{i}+(1-\lambda )x_{j}$$

(2) $$\tilde{y}=\lambda y_{i}+(1-\lambda )y_{j}$$

where $x_{i}$ and $x_{j}$ denote two input samples randomly drawn from the training set, $y_{i}$ and $y_{j}$ are the corresponding labels, and *λ*~Beta (*α*_mix_, *α*_mix_) controls the mixing strength.

The CutMix strategy can be formulated as:

(3) $$\tilde{x}=M⊙x_{i}+(1-M)⊙x_{j}$$

where *M* is a binary mask indicating the replaced region and ⊙ denotes element-wise multiplication.

This strategy dynamically adjusted the mixing strength parameter α and the application probability according to the current training epoch and model accuracy. In the early training stage (epoch < 5), no mixed augmentation was applied. In the warm-up stage (epochs 5–14), MixUp was introduced with α = 0.3 and a probability of 0.3. In the reinforcement learning stage (epochs 15–29), α was increased to 0.5 and the application probability to 0.5. In the reinforcement maintenance stage (epochs 30–44), α was set to 0.4 and the probability to 0.4. In the convergence adjustment stage (epochs 45–59), α was reduced to 0.3 and the probability to 0.3. In the final fine-tuning stage (after 60 epochs), α stabilized at 0.2 with a probability of 0.2. Furthermore, when the testing accuracy reached a high level (>0.92), the system automatically adjusted the mixing parameters to α = 0.1 and probability 0.1 to avoid over-disturbing a converged model. In practical applications, there was a 50% probability that each batch used CutMix (spatial mixing) and a 50% probability that it used MixUp (linear interpolation). This combined strategy strengthened the model’s ability to learn local features while maintaining the integrity of global features. The dynamic adjustment strategy ensured an appropriate augmentation intensity at different stages of model training, preventing instability in the early stage and excessive disturbance when the model approached convergence.

#### 2.6.3. Training Strategy and Optimization

In terms of feature learning, the facial features of Tibetan macaques in Huangshan were not well differentiated, with only subtle differences between individuals. Even the same macaque could exhibit variations in images due to posture and lighting changes. Traditional cross-entropy loss failed to capture such fine-grained feature differences or the correlations between images of the same individual. To address this problem, we adopted the Vision Transformer (ViT) as the backbone network and combined it with supervised contrastive learning (SupCon) to enhance the model’s ability to distinguish subtle features. The self-attention mechanism in ViT enabled the model to capture global dependencies in the image. In addition, a projection head was added after the ViT backbone, mapping the 768-dimensional feature vector through a two-layer fully connected network (768 → 512 → 256) into a 256-dimensional embedding space. The supervised contrastive loss was then applied in this embedding space to pull together representations of the same individual while pushing apart those of different individuals.

Formally, the supervised contrastive loss is defined as:

(4) $$L_{SupCon}=-\sum_{i\in I}\frac{1}{\left|P(i)\right|}\sum_{p\in P(i)}\log\frac{\exp(sim(z_{i},z_{p})/\tau )}{\sum_{a\in A(i)}\exp(sim(z_{i},z_{a})/\tau )}$$

where *z_i_* is the L_2_-normalized projected embedding of sample *i*, sim(*z_i_*, *z_j_*) = *z_i_*^⊤^*z_j_*/(‖*z_i_*‖⋅‖*z_j_*‖) denotes cosine similarity, P(*i*) is the set of indices sharing the same label as sample *i*, A(*i*) = I∖{*i*} is the set of all other samples in the batch, and τ is the temperature parameter (set to 0.07 in this study).

In terms of data balancing, the occurrence frequency of individual Tibetan macaques in the wild was uneven, resulting in unequal amounts of data collected for different individuals. To address the class imbalance issue in the recognition task, the study proposed a multi-level collaborative optimization strategy. At the data sampling level, weighted random sampling was applied, where the sampling weights were inversely proportional to the number of samples in each class, ensuring that the samples from each class in each training batch were relatively balanced. At the loss optimization level, a combination of focal loss (γ = 2.0) and supervised contrastive loss (SupCon Loss) was used. Focal loss automatically reduced the contribution of easy-to-classify samples through a modulation factor, guiding the model to focus on hard and easily confused samples.

The focal loss is defined as:

(5) $$L_{Focal}=-\alpha_{t}(1-p_{t}{)}^{\gamma }\log(p_{t})$$

where *p_t_* is the model’s predicted probability for the ground-truth class, *γ* = 2.0 is the focusing parameter that down-weights the loss for well-classified samples, and *α_t_* is the class-balancing factor.

The overall training objective was formulated as a weighted combination of focal loss and supervised contrastive loss:

(6) $$L_{total}=L_{Focal}+\alpha \cdot L_{SupCon}$$

where *α* is an adaptive weighting coefficient that is dynamically adjusted according to both the training epoch and model performance.

Meanwhile, supervised contrastive loss adopted an adaptive weighting strategy that dynamically adjusted the weight coefficient α according to both the training epoch and model performance. The strategy followed a “warm-up then decay” schedule: in the early exploration stage (epochs 0–9), α was set to 0.05 to allow the model to first establish basic classification capabilities without excessive interference from contrastive learning; in the feature learning reinforcement stage (epochs 10–19), α was increased to 0.08 to strengthen the learning of discriminative feature representations; in the transition stage (epochs 20–29), α was gradually reduced to 0.06 to begin shifting focus toward classification optimization; and in the convergence stage (epochs 30+), α was further decreased to 0.03 to minimize disturbance to the converging model. Additionally, a performance-based adjustment mechanism was implemented: when the test accuracy exceeded 90%, α was automatically reduced to 0.02 to prevent over-regularization of an already well-performing model; when the accuracy was between 80% and 90%, α was capped at 0.05. The weighting coefficient and focal-loss fusion were dynamically adjusted according to the test accuracy to further enhance inter-class separability and intra-class compactness in the feature space. This multi-level balancing mechanism, spanning data input, loss optimization, and feature representation, worked synergistically to effectively mitigate the impact of class imbalance on model performance and provided a robust and efficient training framework for fine-grained individual recognition in wild environments.

In terms of data regularization, we adopted a layered dropout and MixUp/CutMix hybrid strategy. After feature extraction by the Vision Transformer, a feature-level dropout rate of 0.2 was applied, followed by decreasing dropout rates in the multi-layer fully connected classifier (0.3 after the first FC layer and 0.24 after the second FC layer). This progressive design gradually reduced the dropout rate as the feature abstraction level increased, preventing the network from relying excessively on specific feature combinations. In this way, the model was able to fully learn difficult and easily confused features while avoiding overfitting, thereby improving generalization capability while still preserving key discriminative information. At the same time, MixUp and CutMix were alternated with adaptively adjusted parameters. MixUp mixed two samples via linear interpolation, while CutMix enhanced local feature learning through regional replacement.

This prevented the model from depending on a single discriminative cue and encouraged it to integrate multiple complementary sources of information. Together, these methods formed a complementary regularization system that not only augmented the dataset but also effectively suppressed overfitting.

In terms of training optimization, we adopted an adaptive learning-rate scheduling and progressive training strategy. The AdamW optimizer (learning rate 3 × 10^−5^, weight decay 1 × 10^−4^) was used together with a ReduceLROnPlateau scheduler, which automatically adjusted the learning rate according to the F1-score on the test set. When performance stagnated, the learning rate was halved, with the minimum reduced to 1 × 10^−7^. A progressive training strategy was employed. When the test accuracy exceeded 90%, the model automatically switched to fine-tuning mode, during which the learning rate was constrained to remain below 1 × 10^−5^ to prevent excessive updating of the learned features. This dynamic adjustment strategy ensured both rapid convergence and stability in the later stages of training, fully leveraging the advantages of the Vision Transformer in fine-grained recognition tasks.

Specific parameter settings are now provided in Supplementary Tables S2 and S3.

**Figure 2:**
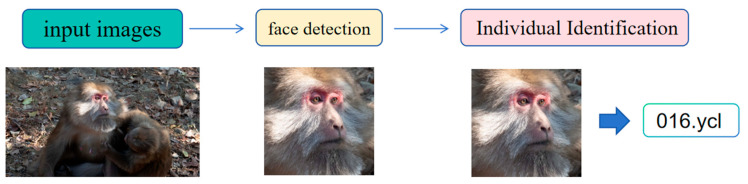
TMacaque-FaceNet Architecture.

The final architecture of TMacaque-FaceNet is shown in Figure 2.

#### 2.6.4. ViT Training Evaluation and Performance Testing of TMacaque-FaceNet

After constructing the model, we trained it on the processed dataset and monitored the training dynamics by plotting loss, accuracy, and F1-score curves on the training and validation sets to verify stable convergence and to detect potential overfitting. We then assessed predictive performance across classes (i.e., different individuals) using the ROC curve and confusion matrix. To further evaluate model performance, we collected an additional independent round of data and constructed a validation set comprising five images per individual. This dataset was fully independent of both the training and test sets, and was extracted from different videos, with efforts made to maximize temporal, event, and visual variability among images. We then applied the trained model to this dataset, recorded its predictions, and calculated the corresponding identification accuracy.

#### 2.6.5. Dimensionality Reduction and Attention Visualization of TMacaque-FaceNet

To further validate the deep discriminative capability of the identification framework proposed in this study and to enhance model interpretability from a mechanistic perspective, dimensionality-reduction-based visualization analyses were conducted. Specifically, t-SNE and UMAP were employed to project samples from different categories into a unified low-dimensional feature space. This visualization strategy enables an intuitive examination of the distribution patterns and inter-class separability in the learned feature space, thereby providing deeper insights into the model’s decision-making behavior. To gain deeper insights into the classification mechanism of our model, we conducted comprehensive attention analysis comprising both visualization and quantitative evaluation. We adopted the Gradient-weighted Attention Rollout method to generate class-specific attention maps, which were subsequently evaluated through Region of Interest (ROI) analysis and insertion experiments to quantify the spatial distribution and discriminative quality of model attention.

#### 2.6.6. Comparison of Established Identification Models

In this study, to ensure that the selected models were well suited to the dataset size, recognition difficulty, and potential environmental disturbances, we combined theoretical considerations with preliminary experiments to screen a range of deep learning models. Ultimately, we selected six baseline deep learning models and further optimized their designs before conducting a comparative evaluation of their performance. Respectively, ResNet152, ViT, Swin-T, ConvNeXt-T, EfficientNetV2-L, and DeiT-S [26,28,29,30,31,32]. All models were trained and evaluated on the same Huangshan Tibetan macaque individual identification dataset to ensure experimental fairness and comparability. Before training, the dataset underwent a standardized preprocessing and data augmentation pipeline, including random rotation, translation, horizontal flipping, and color jittering, followed by normalization. During training, each model was configured with its optimally tuned hyperparameters, such as initial learning rate, batch size, and optimizer. Given the architectural differences among models, hyperparameters were carefully adjusted to achieve the highest possible accuracy for each. The number of training epochs was set to 80, and an early stopping strategy was employed to prevent overfitting and maintain fairness across experiments. The performance of each model was evaluated using two metrics: accuracy and F1-score.

## 3. Results

### 3.1. Training of the Face Detection Module

**Figure 3:**
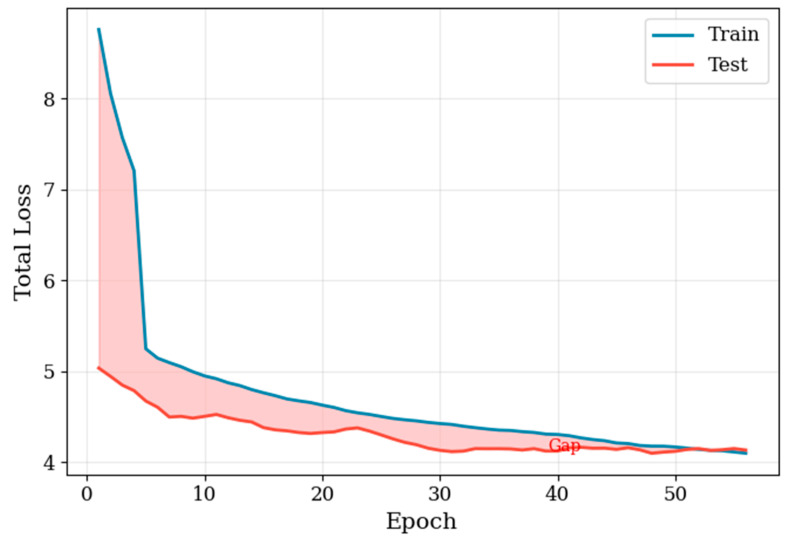
Training loss and test loss curves during training. Both training and test losses show a clear decreasing trend, with the gap between them narrowing as training progresses.

**Figure 4:**
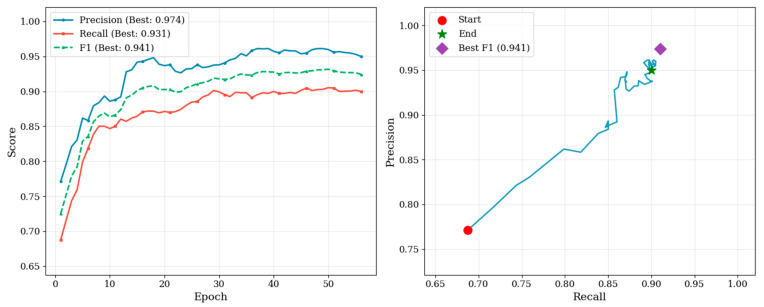
Evolution of precision, recall, and F1 Score during training. Precision, recall, and F1 score increase progressively with the number of epochs. The highest precision reached 0.974, the highest recall reached 0.931, and the best F1 score reached 0.941. The P–R trajectory progressively moves toward the upper-right region, and the optimal operating point achieves the best F1 score of 0.941.

**Figure 5:**
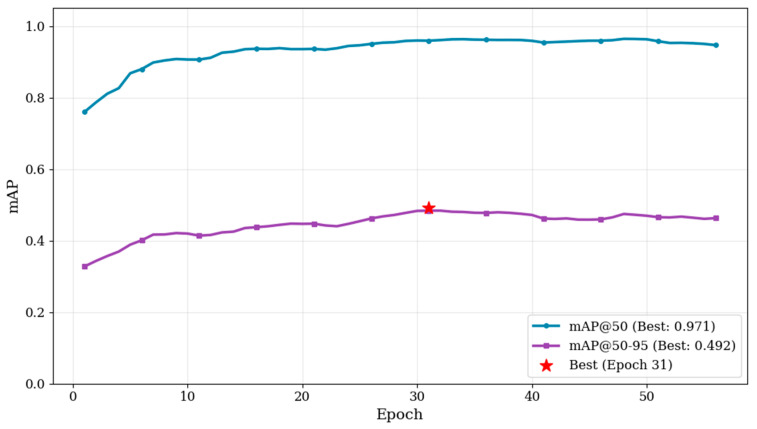
**Evolution of mAP during training.** The mAP curves for both mAP@50 and mAP@50–95 show an increasing trend as training progresses. The mAP@50 reaches its peak value of 0.971, while the mAP@50–95 stabilizes around 0.492.

**Figure 6:**
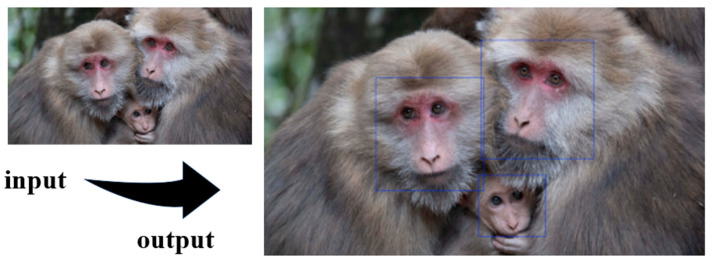
Examples of facial detection. The blue box marks the facial region.

During training of the YOLO-based face detection model, both training and test losses decreased rapidly in the early epochs and gradually converged in the later stages (Figure 3). Precision, recall, F1-score, mAP@0.5, and mAP@0.5:0.95 showed consistent improvements throughout training. The precision–recall curve progressively shifted toward the upper-right region, indicating enhanced detection accuracy and localization performance. The best-performing model achieved precision, recall, and F1-score values of 0.974, 0.931, and 0.941 (Figure 4). The corresponding mAP@0.5 and mAP@0.5:0.95 reached 0.971 and 0.492 (Figure 5). Overall, the optimization process was stable, with no substantial oscillations or evidence of overfitting observed. The input and output results of face detection are shown in Figure 6

### 3.2. Training of the Individual Recognition Module

**Figure 7:**
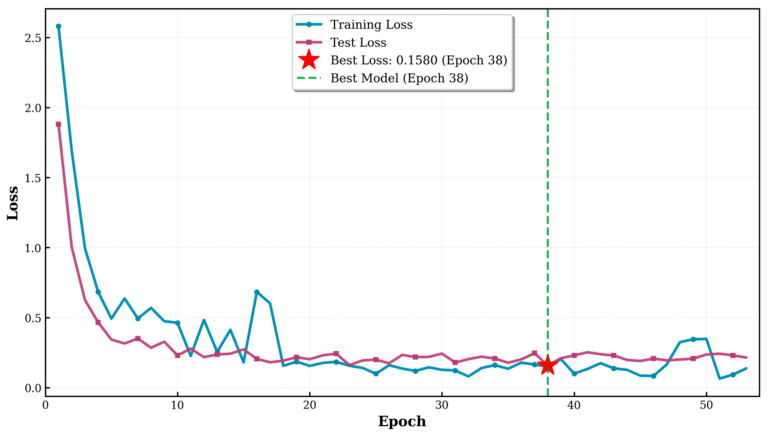
Training loss and test loss curves during ViT training. The training and test losses both show a clear decreasing trend and converge as training progresses. The minimum test loss (0.1580) was achieved at epoch 38, while the final selected model corresponded to epoch 38.

**Figure 8:**
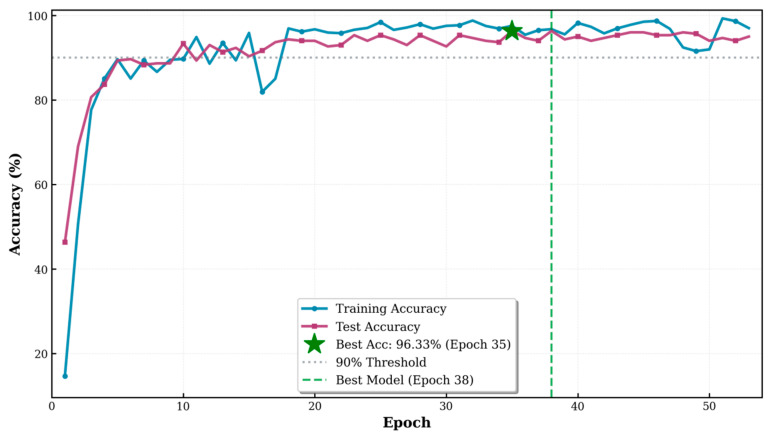
Training and test accuracy during model learning. Both training and test accuracy increased rapidly and then stabilized above 90% throughout training. The highest test accuracy reached 96.33% at epoch 35.

**Figure 9:**
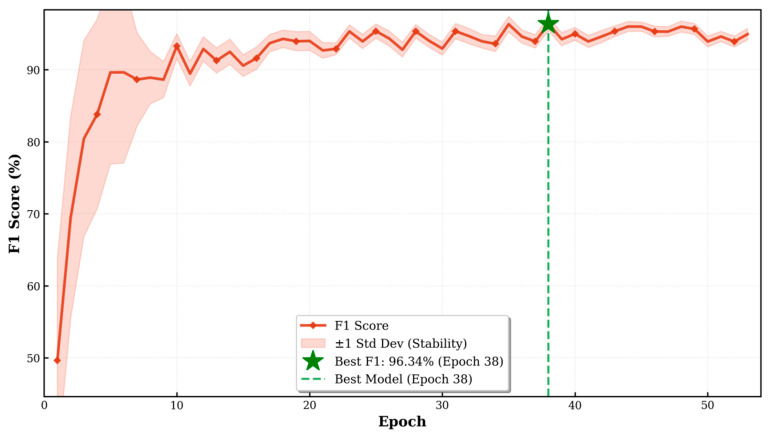
Evolution of test F1 score during training. The test F1 score increased rapidly in the early training phase and then stabilized above 90%, with low variance across epochs. The highest F1 score reached 96.34% at epoch 38.

As shown, both the training loss and test loss rapidly decreased within the first, and then gradually stabilized, maintaining a small gap between them throughout, indicating that the model did not exhibit significant overfitting (Figure 7). The accuracy on the test set stabilized at 96.33% during the later stages of training (Based on the optimal result from 20 independent training runs), demonstrating the model’s strong generalization ability (Figure 8). The F1 score exhibited a similar convergence pattern, ultimately reaching 96.34%, indicating a good balance between precision and recall, effectively addressing potential class imbalance issues in the dataset (Figure 9). Overall, the improved ViT model displayed smooth and robust convergence characteristics, with a minimal performance gap between the training and validation sets, confirming the effectiveness of the proposed improvement strategy.

### 3.3. Performance Testing of TMacaque-FaceNet

**Figure 10:**
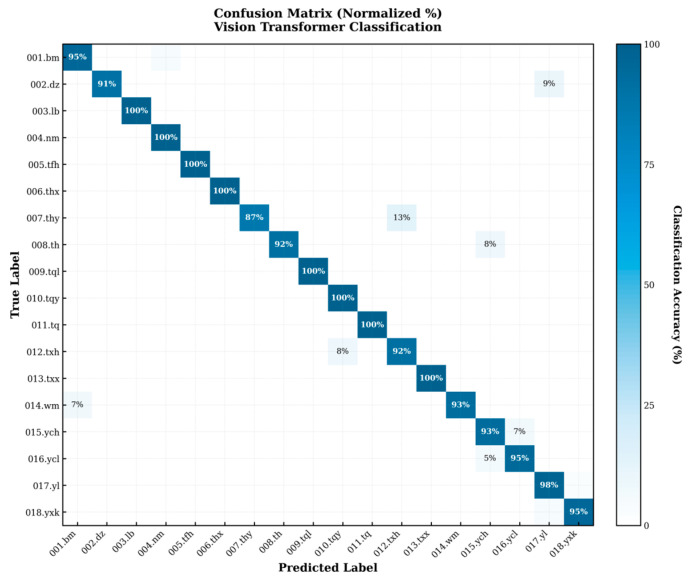
The confusion matrix indicates consistently high classification accuracy across most individuals, with only limited and localized misclassification observed in a small number of cases.

**Figure 11:**
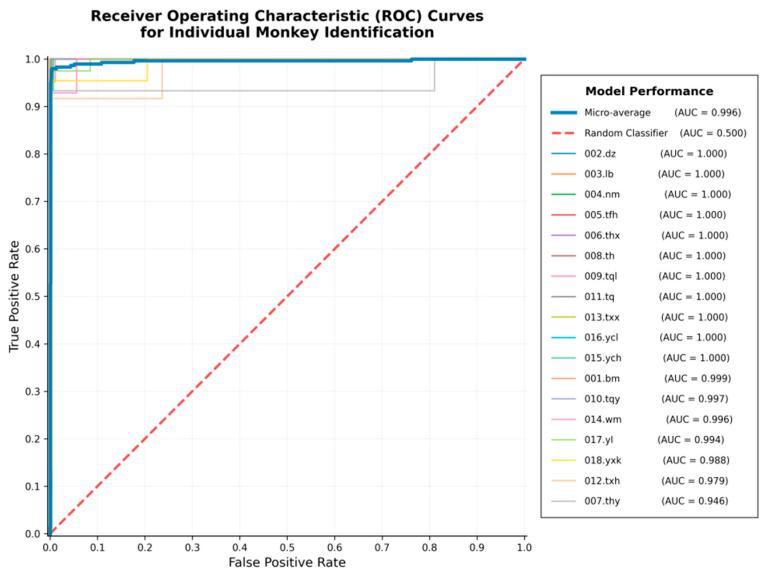
ROC curves for individual-level identification performance. Receiver operating characteristic (ROC) curves for all identified individuals and the micro-averaged model performance. The model achieved consistently high discriminative ability across individuals, with a micro-average AUC of 0.996.

**Figure 12:**
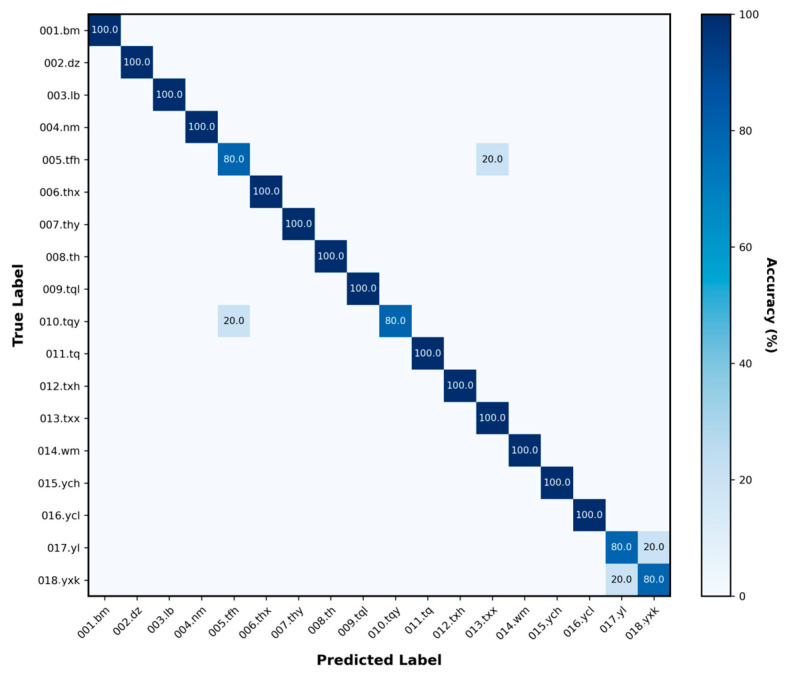
Confusion matrix on new validation set. The model can accurately and effectively identify most individuals.

The confusion matrix directly reflected the model’ s recognition performance, with the majority of individual recognition results distributed along the diagonal and only a few misclassifications occurring between easily confusable individuals (Figure 10). The ROC curve further evaluated the model’ s discriminative ability at the probability level, with a micro-average AUC of 0.996 (Figure 11), indicating high overall discriminative power. On the new validation set, the model also demonstrated strong recognition capability, achieving an accuracy of 95.56% on 90 images, which confirms its robust generalizationability (Figure 12).

### 3.4. Dimensionality Reduction in TMacaque-FaceNet

**Figure 13:**
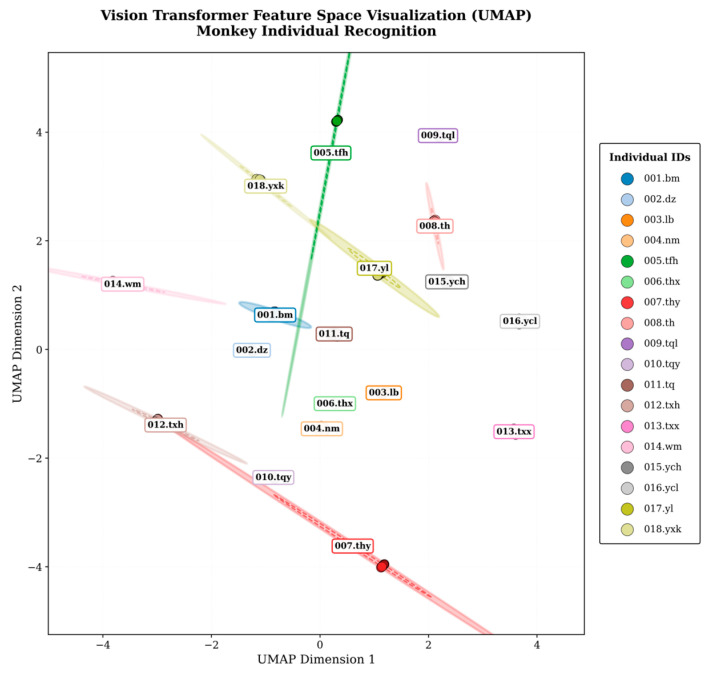
UMAP projection of learned feature representations for individual recognition. The feature embeddings formed compact intra-individual clusters with clear inter-individual separation across nearly all identities. Only a very small degree of overlap was observed.

**Figure 14:**
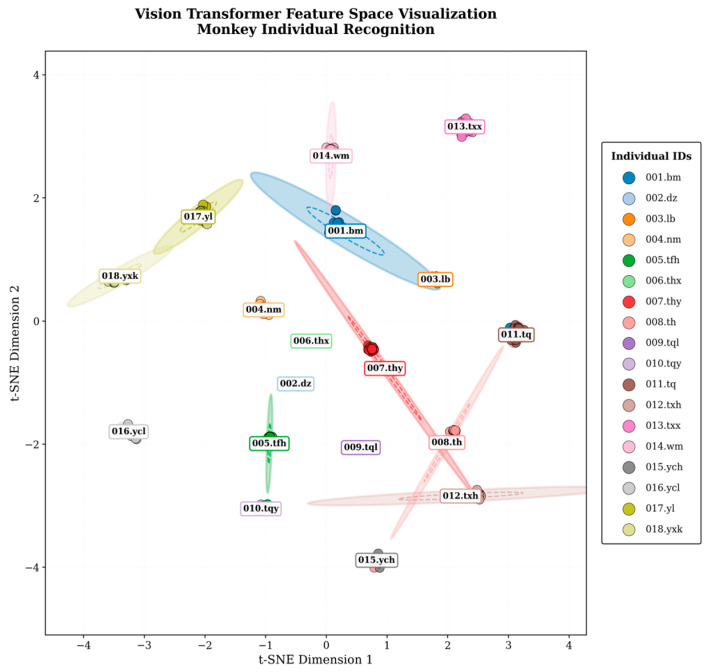
t-SNE projection of learned feature representations for individual recognition. The embeddings exhibit compact intra-individual clustering with clear inter-individual separation across nearly all identities. Only a very small degree of overlap was observed.

After dimensionality reduction and visualization, samples from each individual formed distinct and well-separated clusters in both UMAP and t-SNE projection spaces (Figure 13 and Figure 14). The distributions of different individuals exhibited clear spatial separation, with minimal overlap between their confidence ellipses. These patterns indicate that the model learned discriminative feature representations that organize individuals into distinct regions of the embedding space, consistent with the high classification performance observed.

### 3.5. Attention Visualization and Insertion Experiments of TMacaque-FaceNet

**Figure 15:**
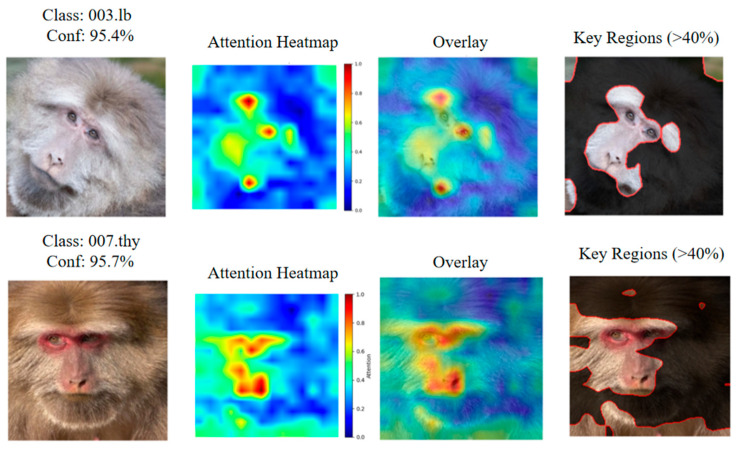
Gradient-weighted Attention Rollout with *γ* = 0.7 applied to a male individual (003.lb) and a female individual (007.thy) shows that the individual recognition model primarily focuses on the center of the face.

**Figure 16:**
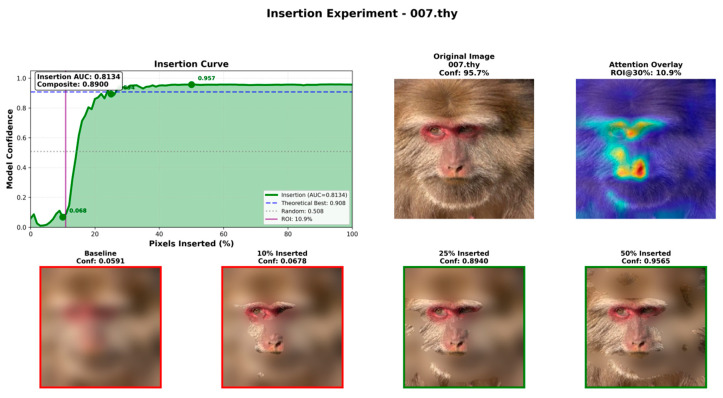
Insertion experiments combined with ROI analysis (*γ* = 1.5) on individual 007.thy achieved an Insertion AUC of 0.8134.

We selected representative images of a male individual (003.lb) and a female individual (007.thy) from the test set for attention analysis. Class-specific attention maps were generated using Gradient-weighted Attention Rollout with *γ* = 0.7. The visualization results demonstrate that model attention is predominantly concentrated on facial regions, with minimal background interference (Figure 15). For quantitative evaluation, we conducted insertion experiments combined with ROI analysis using γ = 1.5, with individual 007.thy as the exemplar. The Insertion AUC reached 0.8134, and classification confidence increased to 89.40% when 25% of pixels (approximately containing nearly complete facial information) were restored, confirming that the insertion of key facial pixels effectively enhances recognition confidence (Figure 16).

### 3.6. Model Selection and Performance

We compared several popular neural network architectures and selected the Vision Transformer (ViT) for Tibetan macaque identification in Huangshan due to its strong interpretability and global modeling capabilities. In terms of performance, ViT achieved 96.33% accuracy and an F1 score of 0.9634 on the test set (Table 3).

## 4. Discussion

In this study, we constructed a small-sample framework for wild Tibetan macaque individual recognition based on manually collected field images and deep-learning models. This system provides a relatively complete identification framework for Tibetan macaque populations. Our results indicate that ViT demonstrated solid performance and promising application potential in Tibetan macaque individual recognition and may offer a transferable methodological reference for future field ecological monitoring and conservation research on rare primate species.,

### 4.1. YOLO Face-Detection Module

From a system-level perspective, the face detector showed stable optimization dynamics. Although it achieved strong performance at mAP@0.5, the comparatively lower mAP@0.5:0.95 (0.492) suggests reduced localization accuracy under stricter IoU thresholds. This is likely related to the ambiguity of monkey facial boundaries, where fur coverage obscures precise contour delineation, as well as unavoidable inconsistencies in manual annotation caused by inter-individual morphological variation. Nevertheless, within the TMacaque-FaceNet framework, face detection primarily serves as a preprocessing step that provides stable facial-region inputs for downstream individual recognition. Its objective is not pixel-level localization accuracy, but robust coarse face localization with good generalization, especially for field samples in which faces occupy only a small portion of the image and background interference is substantial. Under such conditions, reliable target cropping is more practically meaningful than overly precise boundary fitting.

### 4.2. VIT Individual-Identification Module

From the learning curves of loss, accuracy, and F1 score on the training and test sets, the model showed continuous optimization throughout training and gradually stabilized in the later stages. In the early stage, training and test losses decreased rapidly while accuracy increased markedly, indicating that the model quickly captured key facial features. As training progressed, fluctuations appeared in the training curves, likely due to the activation of augmentation strategies such as MixUp and CutMix, which increased task difficulty and reduced the risk of superficial memorization [33,34]. In the later stage, under the combined effects of regularization and learning-rate adjustment, the training and test metrics gradually converged and stabilized.

These results suggest that the training framework used in this study supported stable optimization under small-sample conditions. The absence of obvious overfitting indicates that the model retained a certain degree of generalization rather than simply memorizing the training data. This, in turn, provides indirect support for the effectiveness of the augmentation and optimization strategies in improving robustness under complex field conditions.

### 4.3. Performance Evaluation of TMacaque-FaceNet

To evaluate the performance of the trained model, we conducted a comprehensive assessment of its classification behavior using both a confusion matrix and ROC–AUC curves. ROC–AUC evaluates the model’s discriminative ability in a threshold-independent manner, whereas the confusion matrix reflects the final classification performance under a fixed decision rule. Together, these complementary metrics provide a comprehensive characterization of model behavior in multi-class individual identification tasks.

The confusion matrix indicates that the Vision Transformer achieves high and stable identification accuracy for the majority of individuals, with misclassifications confined to a limited number of individual pairs. Among them, individual 007.thy exhibits a relatively lower identification accuracy (87%), with most errors arising from misclassification as 012.txh. In turn, 012.txh is occasionally misclassified as 010.tqy. In addition, 014.wm and 002.dz are misclassified as 001.bm and 017.yl, respectively, while a clear pattern of mutual confusion is observed between 015.ych and 016.ycl.

Further examination of individual background information reveals that confused individual pairs share pronounced biological similarities. Most misclassified individuals belong to the same sex, which is consistent with previous findings that sex differences in Tibetan macaques are associated with marked variation in facial coloration and texture [23]. In addition, the confusion between 001.bm and 014.wm may be attributed to the fact that both individuals are older males (25 and 17 years old, respectively), corresponding to middle-aged or senescent stages. Age-related facial darkening is a well-documented phenomenon in Tibetan macaques and may increase inter-individual visual similarity [23]. 007.thy and 012.txh exhibit a very high degree of confusion. These individuals share identical sex and age and are both non-immigrants with a degree of kinship, which may further enhance facial resemblance. Similar explanations apply to other confused pairs, such as 002.dz and 016.yl, as well as 015.ych and 016.ycl, which also display high similarity in age and sex structure.

Consistent with these observations, the ROC–AUC curves show that most individuals achieve ROC curves close to the upper-left corner, indicating strong discriminative ability across a wide range of decision thresholds and consistently high true positive rates coupled with low false positive rates [35]. Importantly, individuals with relatively lower AUC values (e.g., 007.thy and 012.txh) correspond closely to those exhibiting poorer performance in the confusion matrix.

Overall, our results suggest that sex and age are key biological factors shaping facial phenotypic variation in Tibetan macaques, while kinship may further amplify visual similarity among individuals. These factors impose inherent constraints on the discriminability of highly similar individuals, thereby limiting model performance in such cases. Future work should aim to elucidate the specific mechanisms by which these biological attributes influence facial phenotype dynamics and to assess their implications for automated individual identification systems.

### 4.4. Dimensionality Reduction and Attention Visualization

To further probe the model’s recognition mechanism, we project the learned embeddings into two dimensions using UMAP and t-SNE [36,37]. The visualisations show that the 18 individuals form well-separated clusters with minimal overlap in the 2D feature space, indicating that the model has learned largely discriminative identity representations [37]. Nevertheless, partial overlap persists among a small subset of individuals with highly similar biological attributes, which is consistent with—and likely explains—the observed misclassifications. In the t-SNE plot, individuals 012.txh, 007.thy, and 008.th do not emerge as fully independent, compact clusters. Complementary evidence from UMAP reveals that their embeddings are elongated and distributed along a similar direction, forming a quasi-continuous structure rather than discrete, separable groups. As a result, when errors occur, the classifier may interpret inter-individual differences as intra-individual variations (e.g., changes in state or degree of the same facial type), leading to confusions among these identities. Overall, these embedding-space analyses align closely with our quantitative performance evaluation, providing additional mechanistic support for the error patterns discussed above.

To further investigate the classification mechanism of the proposed model, we conducted both qualitative visualization and quantitative evaluation of model attention. We adopted the Gradient-weighted Attention Rollout method to generate class-specific attention maps, which integrates the attention rollout strategy proposed by Abnar et al. with the gradient-based weighting scheme introduced by Chefer et al. [38,39]. During post-processing, different gamma correction coefficients were applied for distinct purposes. Specifically, γ = 0.7 was used for visualization to preserve the completeness of the attention distribution, whereas γ = 1.5 was employed for quantitative evaluation to sharpen the attention map and emphasize the most discriminative regions [40]. It should be noted that the choice of different gamma values only affects the visualization characteristics and does not alter the underlying attention distribution.

We performed attention visualization on a large number of test samples and selected one representative female and one male individual for illustration. The results exhibit some deviations from our initial hypothesis. We originally expected the model to focus exclusively on canonical facial landmarks, including the periorbital regions, nose, and mouth. However, empirical observations reveal that although the attention regions largely overlap with the facial area, the most salient attention focuses vary across individuals and even across different facial states of the same individual.

This phenomenon can be attributed to the architectural characteristics of VIT. Unlike CNN, which constructs hierarchical local features, the global self-attention mechanism of ViT enables the model to dynamically select the most discriminative features for each input image [41]. As a result, the model learns data-driven features that are optimized for the classification task, which may differ from human-defined facial landmarks. In the case of individual 003.lb, for example, human experts typically rely on a distinctive nasal defect for identification, whereas the model treats the nasal region as only one of several important attention areas while simultaneously attending to other discriminative features. Such adaptive attention behavior enhances the robustness of the model, allowing it to shift focus to alternative discriminative regions when canonical features are occluded or less salient.

To quantitatively assess attention quality, we conducted ROI analysis and insertion experiments using γ = 1.5, with individual 007.thy serving as an illustrative example. The ROI analysis shows that regions with attention values exceeding the 30% threshold account for only 10.9% of the image area, indicating a highly concentrated attention distribution. In the insertion experiment, starting from a blurred baseline image, original pixels were progressively restored in descending order of attention value. The results demonstrate that the Insertion AUC reaches 0.8134, substantially surpassing the random baseline (0.508). Notably, when only 10% of high-attention pixels were restored, the classification confidence remained low (6.78%), whereas restoring 25% of the pixels—approximately containing nearly complete facial information—led to a sharp increase in confidence to 89.40%.

In short, first, the restoration of facial region information significantly enhances recognition confidence, confirming that the model primarily relies on facial discriminative features rather than being distracted by background information. Second, achieving high confidence requires a sufficient proportion of facial information, suggesting that the model depends on the global relational structure of facial features rather than a simple memorization of isolated local cues, thereby highlighting the advantage of global modeling inherent to VIT.

Compared with previous studies on animal individual identification, our study achieved an accuracy of 96.33% based on 3385 facial images from 18 wild Tibetan macaques (Table 4). This performance is relatively high, although the sample size and total number of images are still smaller than those used in some more established identification systems. For example, a study on giant pandas reported an accuracy of about 95% using around 65,000 facial images from 25 individuals [42]. In contrast, our dataset was smaller and collected under real field conditions, where pose variation, occlusion, unstable lighting, and changes in imaging distance made identification more difficult. Therefore, our results may better reflect the practical potential of the system under complex natural conditions.

Across species, identification performance depends not only on the model itself, but also on the stability of external traits, sampling conditions, and dataset construction. For example, the LemurFaceID system for red-bellied lemurs achieved strong identification performance (98.7%) on 80 individuals using 462 images, partly because this species has relatively clear and distinctive facial patterns and the system used careful image alignment and handcrafted feature extraction [43]. Similarly, a study on Japanese giant salamanders reached an accuracy of 99.86% using 7075 head images from 11 captive individuals [44]. However, the number of individuals to be identified was smaller, the head patterns were more visually distinct, and the images were not affected by complex environmental interference. In contrast, for species with less distinctive facial features and stronger environmental interference, it is more difficult to achieve very high identification accuracy. BearID achieved an accuracy of 83.9% for 132 brown bears using 4674 images [45], while a study on Japanese macaques reported an accuracy of 83% based on 5956 facial images from 42 wild individuals [6]. These findings indicate that, in wild species with less distinctive visual features and stronger environmental interference, achieving very high identification accuracy remains difficult.

In this context, the advancement of TMacaque-FaceNet can be reflected in two main aspects. First, this study focused on wild Tibetan macaques, a primate species of clear value for behavioral ecology research and still achieved high identification accuracy under a small-sample and high-interference setting. This suggests that Tibetan macaque faces contain stable individual-specific information that can be effectively extracted by the model. Second, this study did not rely only on classification accuracy. We also used feature-space visualization and attention analysis, which showed that the model mainly focused on facial regions rather than background cues when making predictions. This improves the interpretability of the framework and increases confidence in its ecological application. Previous studies on chimpanzee identification in the wild and large-scale automatic identification across many primate species have also shown that facial traits can provide a reliable basis for primate individual recognition [20,21]. Our study further extends this idea to wild Tibetan macaques under a small-sample setting.

**Table 4:** Overview of Related Work.

Species	Number of Individuals	Captive/Wild	Identification Area	Image Count	Accuracy (%)	References
Giant panda	25	Captive	face	65,000	95	[42]
(*Ailuropoda melanoleuca*)						
Japanese macaques	42	Wild	face	5956	83	[6]
(*Macaca fuscata*)						
Brown bear	132	Wild	face	4675	83.9	[45]
(*Ursus arctos)*						
Japanese giant salamander	11	Captive	head	7075	99.86	[44]
(*Andrias japonicus*)						
Red-bellied lemurs	80	Wild, Captive	face	462	98.7	[43]
(*Eulemur rubriventer*)						
Tibetan macaques (*Macaca thibetana*)	18	Wild	face	3385	96.33	This study

Our research offers a simple yet effective method for tracking target individuals and acquiring their basic information. By achieving outstanding recognition results at relatively low costs in terms of manpower and resources, we have made it possible for more researchers to build upon this work and conduct field research more efficiently. However, several limitations remain for individual recognition and population monitoring. First, the current system was evaluated under a closed-set setting, in which all individuals were assumed to be known in advance. Although this was appropriate for the proof-of-concept objective of the present study, it limits the applicability of the framework to more complex monitoring scenarios. In future work, we aim to develop a recognition system that can automatically partition datasets based on differences in individual facial features, thereby enabling the automatic recognition of previously unknown individuals. Such an extension would allow the framework to be applied to a wider range of field scenarios and improve its generalizability. Second, although we made every effort to reduce data leakage during dataset construction, uncontrollable factors in field data collection may still result in visually similar images, for example, when the same individual remained in a similar position for a period of time. To reduce the influence of this issue, we incorporated multiple data augmentation strategies with different application probabilities, so that even visually similar images could vary in appearance after transformation. In addition, because data collection was conducted under supplementary feeding conditions, the probability of obtaining frontal, high-quality facial images was likely increased. As a result, the performance of the current framework under non-provisioned conditions or in more spatially dispersed field contexts still requires further evaluation. Finally, the system remains constrained under extreme environmental conditions, such as at night, where poor image quality and blurred facial contours make recognition more difficult. Future work should therefore extend this framework to more open and challenging field conditions so as to improve its recognition ability, adaptability, and practical value for ecological monitoring.

In the future, we plan to integrate existing research results and develop an efficient, automatically updated monitoring system for wild Tibetan macaques. This system will not only support individual recognition but also integrate environmental monitoring data and behavioral analysis, providing a comprehensive ecological monitoring solution. By deploying this system in practical operations, we hope to significantly reduce the workload and costs associated with traditional manual field detection, while improving the sustainability and stability of monitoring efforts. The system will generate real-time dynamic monitoring reports and transmit them to relevant researchers, combining individual recognition with behavioral analysis to provide scientific evidence for the conservation and ecological study of wild Tibetan macaques. As the system continues to be optimized and expanded, we believe this technology will play an increasingly important role in species conservation, ecological monitoring, and environmental assessment, driving ecological protection toward a more intelligent and automated future.

## 5. Conclusions

1. A complete automatic identification framework, TMacaque-FaceNet, was developed for wild Tibetan macaques under natural field conditions. The system integrates face detection and individual recognition and achieved an overall identification accuracy of 96.33% for adult individuals in the YA1 group.

2. The face detection model showed strong robustness in complex natural environments. It could accurately localize facial regions under background complexity, pose variation, and partial occlusion, thereby reducing environmental interference before downstream recognition.

3. The individual recognition model achieved high performance under small-sample and high-interference conditions. It effectively addressed challenges related to limited data, class imbalance, and complex backgrounds, while maintaining strong accuracy and generalization ability.

4. Interpretability analyses confirmed that the model mainly relied on biologically meaningful facial regions for identification. Feature visualization and attention analysis showed clear individual-level separation and limited dependence on background information, supporting the reliability of the recognition mechanism.

5. Overall, TMacaque-FaceNet demonstrates strong potential as a non-invasive tool for long-term monitoring of wild Tibetan macaques. It also provides a useful methodological reference for automatic individual identification in other similar primate species.

## Figures and Tables

**Table 1:** Data Distribution of Facial Images across Individuals.

Sample	ID	Sex	Age	Number of Images
001	bm	male	25	232
002	dz	male	9	123
003	lb	male	9	136
004	nm	male	11	240
005	tfh	female	8	113
006	thx	female	12	84
007	thy	female	15	166
008	th	female	21	134
009	tql	female	11	123
010	tqy	female	8	141
011	tq	male	13	244
012	txh	female	15	139
013	txx	female	16	276
014	wm	male	17	155
015	ych	female	12	170
016	ycl	female	12	214
017	yl	male	10	447
018	yxk	male	11	248

**Table 2:** Scoring Results Evaluated by Experts.

	Fraction 1	Fraction 2	Fraction 3	Average
Round 1	90.0	88.0	88.0	88.7
Round 2	93.0	93.0	95.0	93.7
Round 3	97.0	97.0	97.0	97.0

**Table 3:** Model Comparison.

Model	Best Accuracy (%)	Best F1
TMacaque-FaceNet	0.9633	0.9634
TMacaque-ResNet152	0.9500	0.9493
TMacaque-Swin-T	0.9567	0.9563
TMacaque-ConvNeXt-T	0.9467	0.9468
TMacaque-EfficientNetV2-L	0.9567	0.9567
TMacaque-DeiT-S	0.9500	0.9497

## Data Availability

The datasets used in this study are not publicly available due to privacy and proprietary considerations. Similarly, the source code used for model training is not shared for the same reasons. However, the trained model weights are available for research purposes and can be used to perform inference. The model architecture, input/output format, and inference procedure are fully described in the Section 2, allowing others to apply the model without access to the original training code or data. Upon publication, the trained model weights will be publicly accessible.

## Supplementary Materials

The following supporting information can be downloaded at: https://www.mdpi.com/article/10.3390/ani16071107/s1, Supplementary Table S1. Population structure of theTibetan macaque YA1 group during the study period; Supplementary Table S2 YOLOv8s Model Hyperparameters and Training Configuration; Supplementary Table S3. Vision Transformer Model Hyperparameters and Training Configuration
