# Supplementary material for: TMacaque-FaceNet: Automatic Facial Recognition Based on Vision Transformer for Wild Tibetan Macaques
Source: Animals (Basel). 2026 Apr 3;16(7):1107. doi: 10.3390/ani16071107 (PMC13072414; doi:10.3390/ani16071107)
Supplement: Supplementary file 1 [file animals-16-01107-s001.zip › animals-4214944-supplementary.pdf]

Supplementary Table S1 .Population structure of theTibetan macaque YA1 group during the study period

Male			Female		
**e Group**	**Dominance Rank**	**Individual**	**Age Group**	**Dominance Rank**	**Individual**
**Adults**	1	YL	**Adults**	1	YCH
（**8**）	2	YXK	**(11)**	2	YCL
	3	TQ		3	TXH
	4	WM		4	TQG
	5	NM		5	TQL
	6	LB		6	TH
	7	DZ		7	TXX
	8	BM		8	THY
				9	TQY
				10	THX
				11	TFH

Supplementary Table S2 YOLOv8s Model Hyperparameters and Training Configuration

Parameter	Value
Base Model	YOLOv8s
Input Resolution	1280 × 1280
Epochs	100
Batch Size	16
Frozen Backbone Layers	10
Early Stopping Patience	25
Optimizer	AdamW
Initial Learning Rate	5×10^{-4}
Final LR Ratio	0.01
Cosine LR Schedule	TRUE
Weight Decay	1×10^{-3}
Dropout	0.2
Warmup Epochs	10
Box / Cls / DFL	7.5 / 0.5 / 1.5
Mosaic / Mixup / Copy-Paste	1.0 / 0.2 / 0.1
Rotation / Scale / Shear	15° / 0.4 / 5°
Random Erasing	0.2
Close Mosaic (last N epochs)	15

Supplementary Table S3. Vision Transformer Model Hyperparameters and Training Configuration

Parameter	Value
Model Architecture	Vision Transformer (ViT-B/16)
Input Image Size	224×224 pixels
Number of Classes	18
Feature Dimension	768
Projection Dimension	256
Dropout Rate	0.3
Feature Dropout	0.2
Training Epochs	80
Batch Size	32
Learning Rate	3×10^{-5}
Weight Decay	1×10^{-4}
Minimum Learning Rate	1×10^{-7}
Gradient Clipping	1
Early Stopping Patience	15
Loss Function	Focal Loss + Supervised Contrastive
Focal Loss α	1
Focal Loss γ	2
Label Smoothing	0.05
Contrastive Loss Weight	0.1
Contrastive Temperature	0.07

Supplementary Table S3. (continued)

Parameter	Value
Mixup Alpha	0.4
Mixup Probability	0.5 (from epoch 5)
CutMix Alpha	0.4
Horizontal Flip Probability	0.5
Rotation Range	±10°
Shift Limit	0.08
Scale Limit	0.1
Color Jitter (Brightness/Contrast/Saturation)	0.15
Color Jitter (Hue)	0.05
Gaussian Noise Variance	(5.0, 25.0)
Coarse Dropout (max holes)	8
Optimizer	AdamW
LR Scheduler	ReduceLROnPlateau
Scheduler Factor	0.5
Scheduler Patience	5 epochs
Mixed Precision Training	Enabled
Class-Balanced Sampling	Enabled
Random Seed	42
